# Negative emotions experienced by healthcare staff following medication administration errors: a descriptive study using text-mining and content analysis of incident data

**DOI:** 10.1186/s12913-022-08818-1

**Published:** 2022-12-03

**Authors:** Sanu Mahat, Anne Marie Rafferty, Katri Vehviläinen-Julkunen, Marja Härkänen

**Affiliations:** 1 Department of Nursing Science, University of Eastern Finland, Yliopistonranta 1c, Kuopio, Finland; 2 King’s College London: Florence Nightingale Faculty of Nursing, Midwifery and Palliative Care, James Clerk Maxwell Building, 57 Waterloo Road, SE1 8WA London, UK; 3 Department of Nursing Science, University of Eastern Finland, Kuopio, Yliopistonranta 1, 70210 Finland; 4 Kuopio University Hospital, Puijonlaaksontie 2, 70210 Kuopio, Finland

**Keywords:** Incident report, Medication error, Negative emotions, Support, Nurses, Second victim, Healthcare staff, Text-mining, Content analysis

## Abstract

**Background:**

Medication errors regardless of the degree of patient harm can have a negative emotional impact on the healthcare staff involved. The potential for self-victimization of healthcare staff following medication errors can add to the moral distress of healthcare staff. The stigma associated with errors and their disclosure often haunts healthcare professionals, leading them to question their own professional competence. This paper investigates the negative emotions expressed by healthcare staff in their reported medication administration error incidents along with the immediate responses they received from their seniors and colleagues after the incident.

**Method:**

This is a retrospective study using a qualitative descriptive design and text mining. This study includes free-text descriptions of medication administration error incidents (*n* = 72,390) reported to National Reporting & Learning System in 2016 from England and Wales. Text-mining by SAS text miner and content analysis was used to analyse the data.

**Results:**

Analysis of data led to the extraction of 93 initial codes and two categories i.e., 1) negative emotions expressed by healthcare staff which included 4 sub-categories of feelings: (i) fear; (ii) disturbed; (iii) sadness; (iv) guilt and 2) Immediate response from seniors and colleagues which included 2 sub-categories: (i) Reassurance and support and (ii) Guidance on what to do after an error.

**Conclusion:**

Negative emotions expressed by healthcare staff when reporting medication errors could be a catalyst for learning and system change. However, negative emotions when internalized as fear, guilt, or self-blame, could have a negative impact on the mental health of individuals concerned, reporting culture, and opportunities for learning from the error. Findings from this study, hence, call for future research to investigate the impact of negative emotions on healthcare staff well-being and identify ways to mitigate these in practice.

**Supplementary Information:**

The online version contains supplementary material available at 10.1186/s12913-022-08818-1.

## Background

Medication Errors (MEs) are recognized by the World Health Organization as the leading cause of injury and avoidable harm in healthcare, costing approximately 42 billion dollars annually, which is nearly 1% of total global health expenditure [1]. The safety of patients is at the forefront of the healthcare system; however, healthcare staff can also be traumatized by the aftermath of MEs. Although the healthcare mantra is “first do no harm”, healthcare professionals involved in adverse events can feel guilt, shame, anger, fear, and anxiety [2]. They are often neglected with only a few coping strategies and support systems available to help them [3]. Negative consequences of an adverse event can reach far beyond the “first victim” i.e., the patient. Thus, affecting healthcare staff psychologically making them “second victims” [4]. The term “second victim” was first coined by Dr. Albert Wu to explain the emotions of a young resident who committed an error and had experienced ridicule, shame, and lack of support, from his peers [2]. Although this concept was first applied to physicians, other healthcare staff, including nurses, also experience similar emotions. Scott et al. [5] described the term second victim as “a healthcare provider involved in an unanticipated adverse patient event, medical error and/or a patient-related injury who has become victimized in the sense that the provider is traumatized by the event. Frequently, second victims feel personally responsible for the unexpected patient outcomes and experience as though they have failed their patient, feeling doubts about their clinical skills and knowledge base”[5].

The use of the term second victim has been criticized recently [6, 7] arguing that it might act as a way in which healthcare providers can evade responsibility and accountability and it might be offensive to affected patients and families [6]. Laying accountability at the door of an individual, ignoring the wider organizational ramifications of accountability in terms of the conditions which trigger errors in the first place, can let the organization off the hook. Even though the use of the term “victim” may sound spurious and uncomfortable to many healthcare professionals, patients, and families, it is indubitably an advantage in reinforcing the seriousness and urgency of the problem among policymakers and healthcare managers [8]. Wu et al.[8] have suggested the importance of the use of the term second victim as it is notable and denotes urgency. These assumptions regarding the use of the term second victim are inherent in both positions. Therefore, our research is designed to take this debate one step further by analyzing the consequences of errors in terms of emotional response and lived experiences of healthcare staff.

Regardless of the degree of patient harm, the mere thought of potential patient injury caused by ME is sufficient to induce the feelings of fear, distress, anger, anxiety, guilt and remorse in healthcare staff [9–11]. Although evidence suggests multiple system-based causes of MEs, the error-maker still tends to blame themselves i.e., they should have functioned proficiently [11]. If the seriousness of these issues remains unaddressed, it can negatively affect healthcare workers’ personal and professional well-being causing depression, burnout, Post Traumatic Stress Disorder (PTSD), and even suicidal thoughts [4, 12, 13]. Error prevention has therefore been a focus of major attention for healthcare organizations for years but the impact of MEs on the healthcare professional involved has received less attention. A more nuanced and textured exploration of the impact of the problem upon healthcare workers is required if preventative strategies are to be effective [11].

Previous studies have shown that often MEs causing harm are reported whereas near misses are often under-reported [14]. This underestimates the number of healthcare staff going through negative experiences [15]. Fear of legal consequences, blame, losing patients’ trust, and punishment have been recognized as barriers to ME reporting[16] leading healthcare staff to suffer in silence, sometimes struggling alone in isolation and burdened with a sense of shame [9]. Therefore, a system is needed to mitigate these barriers and create a “just culture guide” which helps healthcare managers to treat staff involved in adverse events fairly, support open and fair culture and maximize learning from errors [17]. However, it is apparent that irrespective of organizational effort in promoting a just and no-blame culture, the stigma persists with respect to speaking up about errors [18].

Patient safety incident reporting has become a common practice, but little is known about the feelings of those who commit or witness incidents. Despite the recent debate regarding the use of the term second victim, we are adopting this terminology throughout our research to analyse the consequences of MEs in terms of psychological responses from healthcare staff. Previous research into second victims has mainly been carried out in a single setting, but this study uses reported incidents at a national level drawing from a range of settings. Also, no previous studies, as far as we are aware, have focused only on Medication Administration Errors (MAEs). To our knowledge, none of these studies have used free-text descriptions of reported medication incidents to review the feelings and emotional responses associated with reporting nor text mining as an innovative method for such analysis.

## Methods

The aim of this study was to investigate negative emotions expressed by healthcare staff in their reported MAE incidents along with the immediate responses they received from their seniors and colleagues after the incident.

### Study design and setting

A retrospective study using qualitative descriptive method and text-mining with an inductive content analysis of the incident data related to Medication Administration (MA) reported in England and Wales was done.

### Description of the data

The data consists of MA incidents (*n* = 72,390) retrieved from the National reporting and Learning System (NRLS) database based on inclusion criteria: (1) incidents reported to have occurred in England and Wales between 1 January and 31 December 2016, (2) medication incident, (3) administration/supply of medicine from a clinical area, and (4) acute National Health Services (NHS) trust (either specialist or non-specialist). The data included incident reports from all levels of healthcare staff ranging from student nurses to senior-level health professionals who were involved in and who have witnessed the MAE incidents.

Data were acquired from NHS England and NHS Improvement. NRLS is largely voluntary and is the only database that includes all types of patient safety incidents. This study used free-text descriptions of the incidents i.e., healthcare staffs’ descriptions of “what has happened?” or “when the incident occurred?” during the medication process.

### Data analysis

**Fig. 1:**
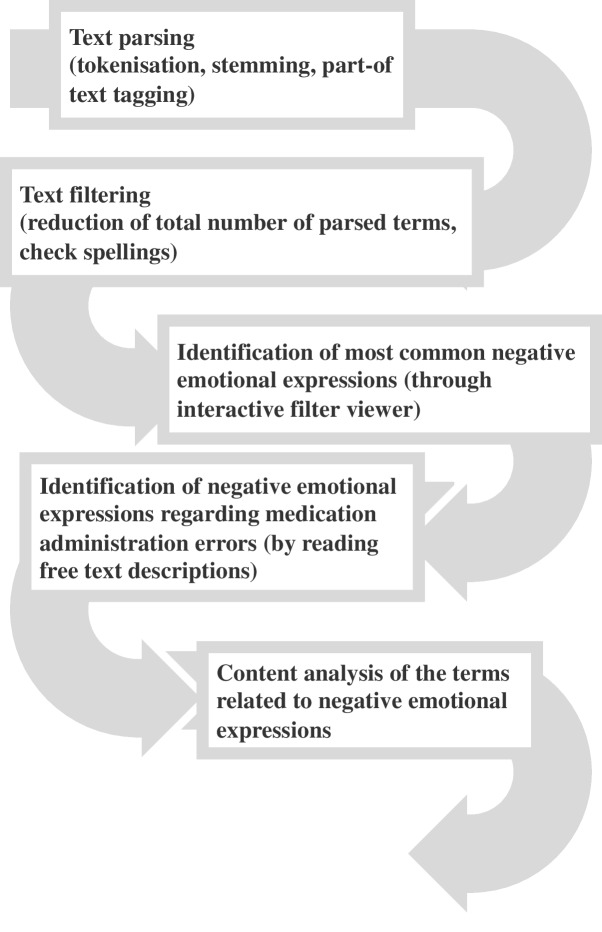
Analysis process of medication administration incident reports’ free text descriptions

First, negative emotional expressions associated with MAEs were defined using the literature and dictionaries (Oxford Learners’ Dictionary, Merriam-Websters’ Dictionary, and Cambridge Dictionary) to define synonyms of the negative emotional expressions (Table 1). Second, those expressions were searched from the free-text descriptions of the incidents which were specifically related to MA. For that, The SAS® Enterprise Miner 13.2 and its Text Miner tool were used. Multiple steps were followed for data analysis as described in Fig. 1. SAS® Text Miner automatically processes the data using ‘text parsing’ i.e., converting unstructured text into a structured form. Text parsing includes tokenization (breaking text into words/terms), stemming (which chops off the end of words reducing words to their stem or root forms), and part-of text tagging (for each word, the algorithm decides whether it is a noun, verb, adjective, adverb, preposition and so on). ‘Text filtering’ was then used to reduce the total number of parsed terms and check the spellings. The English language was chosen for parsing and filtering the text. Using an interactive filter viewer, negative emotional expressions described in the free text were identified and the number of each expression was collected (See Supplementary file 1). For the next phase of the analysis, the most common expressions were chosen which are bolded in online-only material 2 (See Supplementary file 2).

**Table 1:** Common negative emotional expressions associated with medication errors

Negative emotions	Synonyms of the terms [Bold terms: The terms that were marked in bold letters are the ones that were found to be expressed by healthcare staff in their incident reports and thus, are used in the analysis.]
Fear / Anxiety	scared, fear, frightened / frightful / fright, **worry/worried**, terror, anxiety / anxious, nervous, doubt, mistrust, panic, nervousness, afraid, flashbacks, nervous, **stressed**, terrified, petrified, unsettled, on edge, **distressed**, jittery, fidgety, restless, uneasy, dread, apprehension, trepidation, **concerned**, uneasiness
Anger	angry / anger, furious, livid, pissed off, annoyed / annoy, miffed, bitter, enraged, exasperated, fuming, irate, incensed, antagonize, displease, aggravate / aggravation, huff, crossness, bile, spleen, indignation, displeasure, exasperation, dudgeon, distress, sullen, sulky
Disturbed	**agitated**, shock, troubled, unsettled, nervous, restless, shake, discompose, unhinge, stunned, choked, unbalanced, uneasy, solicitous, **upset**, uptight
Sad	sad, sorrowful, unhappy, **sorry**, mournful, rueful, stressing, lugubrious, woeful, afflicted, woe some, wretched, miserable, nasty, lousy, crappy
Shame	shame / ashamed, disgrace, ignominy, stigma, mortification / mortified, reproach, dishonor, dishonour, indignity, discredit, obloquy, embarrassed, shamefaced, hangdog, self-conscious
Guilt	guilt / guiltiness, self-blame, regret / regretful, offense, **fault**, failing, culpability, self-reproach, blameworthiness, wrongdoing, misconduct, self-reproach, self-condemnation, remorse, remorsefulness / remorseful, contrition, contriteness, compunction
Self-esteem	self-worth, self-regard, self-respect, self-integrity, self-confidence, self-disappointment, incompetent
Depression	depressed, frustrated, overwhelmed, devastated, hopelessness, sleeplessness, crestfallen, hump

References (The references mentioned along with this table are the reference list used to extract the synonyms for negative emotions.)

Anger. *Oxford Learners Dictionary*, https://www.oxfordlearnersdictionaries.com/definition/english/anger_1?q=Anger

Disturbed. *Merriam-Webster Dictionary.* https://www.merriam-webster.com/thesaurus/disturbed

Fear. *Merriam-Webster Dictionary*. https://www.merriam-webster.com/dictionary/fear

Jones JH, Treiber LA. More than 1 million potential second victims: How many could nursing education prevent? Nurs Educ 2018; 43:154-7.

Jones JH, Treiber LA. When nurses become the “second” victim. Nurs Forum 2012; 47: 286–291.

Krzan KD, Merandi J, Morvay S, Mirtallo J. Implementation of a “second victim” program in a pediatric hospital. Am J Health Syst Pharm 2015; 72:563-7.

Sad. *Merriam-Webster Dictionary.* https://www.merriam-webster.com/thesaurus/sad

Stillwater AR. Medication errors: the school nurse as second victim. NASN Sch Nurse 2018; 33:163-6.

Treiber LA, Jones JH. After the medication error: Recent nursing graduates’ reflections on adequacy of education. J Nurs Educ 2018; 57:275 − 80.

Treiber LA, Jones JH. Making an infusion error. J Infus Nurs 2018; 41:156 − 63.

Expressions chosen for analysis were used as a search term in an interactive filter viewer. All the descriptions of the incidents that included those expressions (a total of 1861 incident reports) were collected and read through repeatedly. In the first phase of this analysis, the aim was to define who had experienced the emotional feeling. Most of negative emotions were expressed by patients or relatives (See Supplementary file 1). Those descriptions of incidents that included negative emotions expressed by healthcare staff and which were expressed in relation to MAEs (*n* = 93) were then selected for further analysis.

Content analysis was used to analyze the data. The lead author followed an inductive content analysis where the researchers carefully read, organized, and integrated and formed categories, concepts, and themes by comparing the similarities and differences between the coded data [19]. The lead author read through the data repeatedly and during this process, identified the main theme which is: Emotional expressions of healthcare staff after MAEs. The data were organized into main themes and sub-themes. After the preliminary classification, a co-coder [the last author of this paper] participated in the analysis and read the classification structure and the related data independently. Once thematic saturation was achieved, both researchers analyzed the entire data corpus according to standard thematic analysis techniques [20]. All authors contributed to the final form of the analysis. Finally, direct quotes were used to support the findings.

## Results

### Negative emotional expressions of healthcare staff after MAEs

We found 15 different types of negative emotional expressions used including worry, anxiety, annoyance, agitation, stress, unhappiness, distress, concern, anger, upset, shock, sorry, fault, depression, and frustration. These 15 different types of emotions were expressed 1,861 times in the incident reports (See Supplementary file 1).

**Fig. 2:**
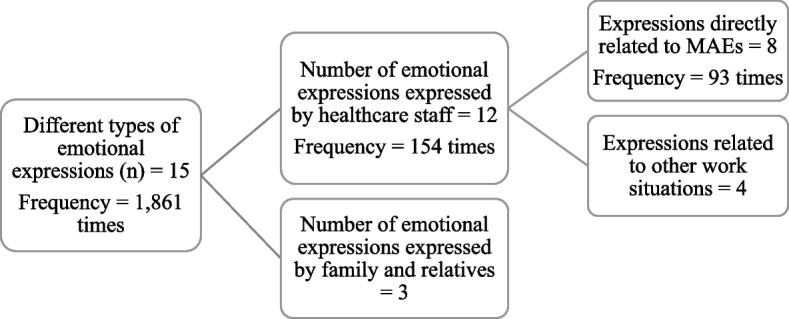
Typology and frequency of emotional expressions

Among those emotional expressions, 12 were exhibited by the healthcare staff and were mentioned 154 times. Only eight of those 12 expressions: worry, upset, agitation, faulty, sorry, concerned, stressed, and distress were expressed by healthcare staff in direct relation to MAEs, the frequency of expression here was 93 times. The data extraction process in presented as a flowchart in Fig. 2.

The key emotions revealed were further classified into four categories: (1) feeling of fear, (2) feeling of upset, (3) feeling of sadness, and (4) feeling of guilt (Table 2).

**Table 2:** Category and sub-categories of negative emotions

Category	Sub-category
Negative emotions expressed by healthcare staff after medication administration errors	Feeling of fear: *Stressed = expressed for 3 times* *Worried = expressed for 11 times* *Concerned = expressed for 23 times* *Distressed = expressed for 3 times*
	Feeling disturbed *Upset = expressed for 24 times* *Agitated = expressed for 2 times*
	Feeling of sadness: *Sorry = expressed for 13 times*
	Feeling of guilt: *Fault = expressed for 14 times*

### Feeling of fear

Healthcare staff described their feeling of fear regarding MAEs using four different synonyms i.e., distressed, concerned, stressed, and worried. Staff mentioned how fearful they were when they discovered their mistakes. Distress was revealed in three of the incident reports as expressions of fear of healthcare staff. Usually, MAE incidents were reported either by the error-makers themselves or by those witnessing their errors. One of the staff described the fear felt by her colleague (staff nurse) by reporting how distressed he was after he administered a medication through wrong route (intravenous instead of oral):

“*I was assessing a patient on Ward X when a staff nurse approached me extremely distressed and agitated. He then ran into the utility without explaining what the problem was. I followed him…nurses were present who proceeded to explain that the nurse who approached me had given a patient 2mls of Oramorph [liquid morphine that has to be given orally] intravenously*…"

Healthcare staff also expressed the extreme pressure which acted as an important contextual trigger, driving intense the feelings of fear. Another emotion linked to fear was “concerned” which was expressed in 23 cases by healthcare staff after making an error. One of the healthcare staff reported an error (prescribed wrong strength), which the staff realized two hours later and became concerned about it:

"*Prescribed TTA (to take away) of ‘Augmentin [Amoxicillin Clavulanate] Duo’125/31 8 ml TDS [three times a day]. As written, this would be a drug error-there is no 125/31 strength of …This was my error, which I realized and became concerned about 2 hours later*…"

Stress was expressed in three cases by healthcare staff while reporting the incident; however, this emotion was expressed by staff not as their feelings after MAEs, but as the reason underlying MAEs. These kinds of explanations were found in many incident reports where healthcare staff accepted the error but eventually pointed towards other hidden causes behind the error:

"*Gave Clexane [Enoxaparin] 60 mg to wrong patient. Ward extremely busy- heavy workload and was very stressed due to workload*…"

Being “worried” was another expression of fear reported in 11 incident reports by healthcare staff. They were found to be worried about several situations such as the health of patient, degree of harm caused by error, associated legal procedures, and their professional career. One staff nurse was worried about the patients’ condition as he did not administer insulin dosage to one of his patients:

"*Staff nurse came to me at the end of the shift and stated that he thought that the patients’ insulin was prescribed prn [whenever necessary] and had not given any…I explained he needed to inform the nurse in charge…he was very sincere and worried that he had not given this insulin*…"

### Feeling disturbed

The feeling of being disturbed was expressed using two synonyms: upset and agitated. They addressed themselves as being upset in 24 incident reports following MAEs committed either by themselves or by their fellow staff. Healthcare staff reported the error made by fellow staff member and described the emotion of his/her colleague as:

"*Nurse called me was very upset to explain that she had given wrong treatment to patient*…"

Even near miss situations have caused healthcare staff to get emotionally disturbed. Even after apologizing with patient and family, healthcare staff felt upset thinking that if they were not aware of the near miss situation in time, patients’ condition would have been severe:

"*SN asked me to do a syringe driver with her for a palliative patient…on drawing up the ketamine driver, myself and SN made a drug error in which we drew 5 times more ketamine than the required dose…The family and patient have been informed of the drug error we made and we gave our sincere apology for our faults…both myself and SN are very upset with the near miss situation and aware that things could have gone very differently*…"

Healthcare staff expressed being agitated in two reports after discovering that they had committed MAEs, except in some situations, where staff though agitated denied their mistake by underestimating the severity of the error they made:

"*Patient was discharged off the system by the nurse without confirming with medical team/pharmacy that patient was ready to go… Patient left without anti-sickness medication which the team had told her she could have…Nurse was evidently agitated that the incident was being reported and did not understand that she should check with the team before authorizing*…"

Some reports revealed extreme negative emotions associated with feelings of upset such as being devastated and questioning one’s own professional competence. The use of such intense and traumatic language can reflect how much the healthcare staff were impacted and even emotionally wrecked after MAE. One healthcare staff after accidentally administering wrong dosage to the patient, reported that the error was entirely his/her own fault:

"*Pt px 120 mg on gentamicin on EOMA, I accidentally gave 210 mg in error. This was entirely my fault …The checker confirmed what I had done. I am so devastated about this and really upset I’d made such a mistake…today was just hectic and I lost concentration*..."

### Feeling of sadness

Healthcare staff expressed their feeling of sadness at being sorry for the mistake they had made; it was one of the most common negative emotional expressions expressed in 13 cases. Most staff used this to express a sense of remorse after the error. After missing a dose of insulin for a patient, one healthcare staff expressed his/her sadness by stating that he/she is sorry about the incident:

"*I am sorry to say that I missed one dose of insulin (at 22.30…) for one of my patients*…"

Along with the feeling of sadness, one healthcare staff also mentioned about learning from the error and how he/she have accepted that she was wrong to assume things:

"*I was sitting at the desk, staff nurse handed me a tray with intravenous antibiotics and said, here is one because I had given her patient drug chart, I assume it was patients’ medication. I did not take the drug chart with me to the patient and afterwards when staff nurse came with patients’ drug, I realized I have given the wrong drug. I was very upset as I have never done anything in this form before. I always take the drug chart with me to the patient. I am deeply sorry, and this is a massive learning curve for me, I hold my hand up it was wrong to assume this*."

Healthcare staff who had mentioned learning from the error was quite common in many incident reports. However, there were few cases where the staff did not understand the seriousness of the error she has caused:

"…*I spoke to the student nurse about the seriousness of her actions, she said sorry; however, I did not feel she understood the seriousness of what she did*…"

### Feeling of guilt

In 14 incident reporting cases, healthcare staff were aware of their mistakes and the consequences they might have. They expressed their guilt and identified themselves as being at fault and blaming themselves.

"*IV flucloxacillin drawn up and checked by myself and staff nurse…administered drug however in error name band/ allergy band not checked. Realized immediately after administration that I had gone to the wrong patient and given the incorrect medication…conversation with senior staff nurse about error. Explained that the error was my fault completely…patient does not appear to have come to any harm*…"

However, this emotion was not just expressed following the error, but also as another reason for error attribution. For example, in the report below, a staff member made an error, and blamed herself and phone reception for being muffled:

"*I had to hand over two diabetic patients to the 5–8 pm. I rang Ward sister and confirmed this again later. However, patient was not reallocated, and insulin omitted…Ward sister apologized for yesterday missed patient…she said the reception to her phone was muffled and that it was her fault*…"

#### Immediate response from seniors and colleagues

Some of the healthcare staff while reporting their feelings behind MAE incidents also discussed regarding the immediate responses they received from their seniors and colleagues. Healthcare staff explained how their seniors and colleagues responded after they were informed about MAEs. These responses are categorized into two sub-categories: (1) Reassurance and support and (2) Guidance on what to do after an error.

#### Reassurance and support

In three incident reports, healthcare staff mentioned about the reassurance and positive support they received from their seniors and colleagues after the disclosure of MAEs, about how they tried to handle the situation very calmly without getting angry. This helped them to cope effectively without undue stress and burden. A nurse mentioned that she reassured one of her colleagues who was very disturbed after she gave the wrong medication to her patient:

"*Staff nurse by mistake gave the patient wrong medication…. misread the information by being interrupted by a patient and member of staff…. I reassured the staff nurse as she was very upset*…"

Even a little support and reassurance and few kind words during the time of MAEs can help the healthcare staff to cope up with the situation effectively. As one member remarked:

"*Medication error – digoxin prescribed in two doses (125mcg and 62.5mcg) did not realize and administered…Immediately alerted sister in-charge of ward and contacted doctor. Doctor did not come to the ward but was happy that observations had been recorded…and told us not to worry*…"

#### Guidance on what to do after error

In 11 incident reports, healthcare staff mentioned about receiving advice from their seniors and colleagues regarding the right thing to do after making an error. They have been guided to observe the situation of the patient to ensure that no serious harm would be caused to them:

"…*Administered the oramorph in an unlabeled syringe which was in the same tray as a 10ml flush…I discussed the situation with the medical registrar on call who advised me to monitor observations regularly*…"

"…*I spoke to the nurse in charge after the error from the following shift who said that I should speak to the ward manager at the earliest opportunity which I did*…"

Furthermore, in cases where healthcare staff neglected to document the incident, a colleague intervened to guide the staff member to follow the protocol. As one staff member described:

"…*I discussed the incident with a colleague shortly afterwards. However, I neglected to escalate and correctly document the incident…The aforementioned colleague has since approached me to discuss the incident, further to this I approached and discussed the incident with my ward manager*…"

## Discussion

Our study identified four categories of negative emotions expressed in incident reports: feelings of fear, disturbed, sadness, and guilt with various sub-categories. In addition, this study also captured the immediate responses received by healthcare staff after they informed their seniors and colleagues about MAEs including the reassurance, support, and guidance on what to do after an error. Incident reporting by healthcare staff in this study indicated that unintentional harm caused due to MAEs and even near misses can affect the healthcare staff involved in error emotionally, increasing their risk of becoming the second victim of MAEs, confirming previous research [9, 21].

A major finding of this study was the negative emotions experienced by healthcare staff after MAEs. Healthcare staff in this study expressed their fear while reporting incidents by using negative emotions such as stressed, distressed, concerned, and worried. They not only blamed themselves for these mistakes, but also considered other additional explanations which, they perceived as causing the error. These kinds of emotions can be related to staff members’ narration of fear and anxiety for patients’ well-being and for their own professional careers [22]. Similarly, feelings of being disturbed expressed as being upset and agitated were widely mentioned in incident reports. Identical reasons such as realization of the error and thoughts of the possible seriousness of the error and associated issues lay behind emotions. Further, feeling of sadness expressed as being sorry for the mistake made was another most common emotional expression. Also, healthcare staff felt a deep burden of responsibility for their actions. Feelings of being guilty or at fault is one of the risk factors for healthcare staff for becoming the second victim of MEs. It can also cause loss of self-esteem and inculcate a sense of failure and hopelessness. In a similar study by Treiber & Jones [22], nurses, upon committing even minor errors, expressed raw and painful emotions, regardless of the degree of harm. Nurses can often recall the details of the error and what they felt at that time [22]. While the lack of any apparent linkage between emotional response and degree of patient harm might appear counter intuitive, one possible explanation might be that healthcare professionals are not well enough supported by their organizations to cope with any form of negative experience. Thus, those affected might develop strong negative emotion [23].

Making an error might also have serious consequences for disrupting the personal and professional lives of staff, causing personal and moral distress, and affecting the quality and safety of patient care [23]. It is crucial to pay attention to these emotional expressions as incidents that are sensitive and make an impact, are often remembered, and reflected in the attempt to prevent recurrence. On the other hand, these incidents can unintentionally impose a mental burden on healthcare staff making them second victim [2]. Our findings confirms that MAEs can generate negative feelings in healthcare staff associated with it, which can endure long beyond the immediate effect.

Research has confirmed a direct relationship between nurse staffing and missed patient care [24, 25], revealing poor nurse staffing as a risk factor for MEs along with other organizational factors such as poor working conditions, distractions, and high workload [26]. Similarly, in this study, reporters mentioned their own actions as a trigger for MAEs along with the above-mentioned factors whereas some reporters explained organizational and environmental conditions and context surrounding the error as reasons to reduce blame. In the absence of support, self-blame seems to assume greater prominence. This can have long-term repercussions for maintaining emotional health and well-being, a major failure of workforce strategy, especially during the pandemic situations.

The current study also found other healthcare authorities responding in several ways after being informed about MAEs. Sometimes, staff may not know what to do after MEs, they might panic and lose control. Thus, adequate support from colleagues and seniors sensitive to these issues may prevent the error-makers from translating further into second victimhood of MEs. How the organization and related individuals responds is clearly linked to the emotional impact the error can have on the healthcare staff who made the error. Appropriate support and guidance from seniors and colleagues have been found to alleviate the suffering, while lack of support has increased their psychological burden [27]. Some of the healthcare professionals in our study also opted for consulting with their seniors: doctors, colleagues, and mentors after MAEs and reported about how they have received guidance and suggestions, which helped them to cope effectively. Emotional support plays a vital role in restoring faith and confidence among healthcare professionals in patient safety. Support from co-workers and healthcare institution helps the error-makers to retain a sense of control [2]. Reassurance from seniors and colleagues can also strengthen healthcare staff’s self-esteem and facilitate the correct reporting of MAEs. As is well known, only a fraction of incidents are reported thus deterring the improvement of patient safety with barriers identified as time pressure, fear of the consequences [28], poor institutional support, lack of feedback, a blame culture, and inadequate training [15]. Yet, we can still improve patient safety by identifying these barriers. Moreover, while some staff members perhaps too readily assumed responsibility for errors, as reflected in the prominence of self-blame, others demonstrated reluctance, which could be linked to fear of the consequences of MAEs. Furthermore, little is known about the dynamics and consequences of reporting-what prompts some to report and others not to do so. We demonstrate that the emotional expression of staff can be extremely distressing and negatively impact health and well-being of healthcare staff.

### Implications for practice

Our findings indicate that immediate negative feelings experienced by healthcare staff after making MAEs can have long-lasting impacts that stretch far beyond the event itself thus potentially traumatizing them and inducing ruminative thoughts, which trigger the memory. The short, medium, and long-term consequences of errors are unknown as yet but could contribute to burnout and other factors associated with intention to leave the profession. Indeed, a negative memory that will stay with them forever, if not handled accurately. They could potentially become second victims of an error, if unable to confront and deal with negative feelings associated with the error. One source of challenge could be stigma related to this making it difficult to continue to work after MAE. Our findings suggest appropriate guidance and support from fellow staff members could help healthcare staff to handle the situation effectively. Therefore, it should be paramount to tailor appropriate support from persons in-charge and colleagues and to promote an open culture where it is understood. Errors can impair mental health of those who are involved, hence, the system triggers surrounding such errors need to be understood and prevented. In addition, more detailed information about these emotions after incidents and their long-term consequences on emotional well-being should be studied in future.

### Implications for research

The negative feelings expressed by healthcare staff after MAEs identified in this study could provide the basis for designing an intervention study to support emotionally affected staff in healthcare institutions. It could be helpful to design a support program which recognizes the importance of expressed emotion and its consequences for internationalizing a sense of self blame and victimhood and the long-term repercussions this might have for the mental health and well-being of the health workforce.

### Strengths and Limitations

As far as we are aware, this is the first-time text-mining and content analysis have been used to identify negative emotions reported by healthcare staffs’ MAEs, derived from free text in a large national database. A text-mining approach was used for identifying reports that included emotional expressions, as manual data analysis would have been almost impossible for such a big data set and this approach has been recognized to be time-effective in analysing big-data regarding medication incidents (Härkänen et al., 2019). Further, the emotional expressions identified in this study are relatively rare. These descriptive data of emotional expressions nevertheless cast light on the issues related to MAEs. Furthermore, the researchers adhered to the Standards for Reporting Qualitative Research (SRQR) checklist (see the list in Supplementary file 3).

However, while analyzing the free-text descriptions, we may have missed some important expressions as this was a pilot methodology we were testing, subjective decisions were made. Similarly, it was very difficult to combine the synonyms of the word used to express the negative emotions which can give rise to ambiguities. For example, in many cases, one single word could either be a verb, or noun or an adjective i.e., words can have different implication [29]. On the contrary, this study sheds some light upon how important it is to write incident report and to identify the negative emotions of staff, to prevent further consequences from occurring, encourage reporting and put support mechanisms in place. Patient safety incident data is likely to contain some limitations, more specifically, reporting error and bias which will affect the number, type and temporality of reported incidents and data interpretation [30]. Since reporting is largely voluntary, there are some potential limitations of NRLS being a reliable indicator of exact number of incidents. Nevertheless, increasing number of incidents may reflect an improved reporting culture. Further, the methodology did not allow for the identification of any positive emotions that might have been expressed by healthcare staff when reporting MAE incidents, as only free-text descriptions which included negative emotions were analyzed .From the free text-descriptions, most of the reports were found to be from nurses, however, staff-specific generalizability and scope is limited due to lack of staff type identification in NRLS data i.e., ST01 [31]. This makes it difficult to precisely quantify the impact and potential benefits of this research.

## Conclusion

A wide range of negative emotions was expressed by healthcare staff after reported MA incidents. However, the associated psychological trauma and low mood expressed by healthcare staff represent significant negative impacts underlying reported negative emotions. It is more likely that MAE incidents are under-reported, therefore problems could be much higher in terms of prevalence and magnitude. There was tremendous variation in reports of healthcare staff encountering with MAEs; some reacted in extremely negative ways, whereas the majority expressed little about their feelings. Although many of the incident reporters did not express their feelings in their reports, there is also the possibility of them being affected by the aftermath of MAEs. Several actions were taken by healthcare staff to help cope with the error: which included, seeking guidance, reassuring, and supporting each other. This calls for further efforts from healthcare organizations to support healthcare staff as a matter of routine when encouraging reporting. Though we do know little about the long-term consequences, from what we see in our data, the scarring effect could potentially be considerable. Therefore, support programs need to be co-designed but incentivize to reward reporting without imposing an emotional burden on already overburdened staff. This is vital for error reporting, safety, and ultimately prevention to flourish in the long run. First and foremost, the system needs to promote psychological safety for its users, which our research currently demonstrates.

## Supplementary Information

**Additional file 1: Supplementary file 1. **Number of incident reports with negative emotional expressions and description about the healthcare staffs’ feeling. **Supplementary file 2. **Number of negative emotional expressions related specifically to medication administration incident reports (*n*=72,390). **Supplementary file 3. **SRQR checklist for reporting qualitative studies.

## Data Availability

Data supporting the findings of this study are made available from NRLS/NHS Improvement. However, restrictions apply to the availability of these data. For this current study, these data were used under license, therefore, are not publicly available. Data are however available if contacted to authors (MH, AMR, SM) upon reasonable request and with permission from NRLS/NHS Improvement.

## Abbreviations

ME: Medication Error
MAE: Medication Administration Error
MA: Medication Administration
NRLS: National Reporting and Learning System
NHS: National Health Services
SRQR: Standards for Reporting Qualitative Research
