# Supplementary material for: Negative emotions experienced by healthcare staff following medication administration errors: a descriptive study using text-mining and content analysis of incident data
Source: BMC Health Serv Res. 2022 Dec 3;22:1474. doi: 10.1186/s12913-022-08818-1 (PMC9719256; doi:10.1186/s12913-022-08818-1)
Supplement: Supplementary file 1 — Additional file 1: Supplementary file 1. Number of incident reports with negative emotional expressions and description about the healthcare staffs’ feeling. Supplementary file 2. Number of negative emotional expressions related specifically to medication administration incident reports (n=72,390). Supplementary file 3. SRQR checklist for reporting qualitative studies. [file 12913_2022_8818_MOESM1_ESM.docx]

**Supplementary file_1.** Number of incident reports with negative emotional expressions and description about the healthcare staffs’ feeling

| **Negative emotions** | **Synonyms of the terms** | **Number of incident reports with expressions** | **Who had the feeling?** |
| --- | --- | --- | --- |
| **Fear / anxiety** | **Worry + Worried** | 33 + 99 | Staff about patients’ health x 19  Staff not worried x 1  **Staff worried after MAE x 11** |
|  | **Anxiety + Anxious** | 59 + 124 | **Staff anxious after MAE x 0** |
|  | **Stress** / -**ed** (verb) + S**tress** (noun) | 47 + 30 | Staff stressed x 7  **Staff stressed after MAE x 3** |
|  | **Distressed** | 104 | Staff distressed with other situations x 4  **Staff distressed after MAE x 3** |
|  | **Concerned** | 209 | Medical staff not x 118  Other ward staff after an event of ME x 109  Staff about patient’s health x 74  **Staff after commencing MAE x 23** |
| **Anger** | Angry | 91 | **Staff member x 5**  **Staff after commencing MAE x 0** |
|  | Annoyed | 23 | **Staff member x 6**  **Staff after commencing MAE x 0** |
| **Disturbance** | **Upset** | 325 | **Staff member x 35**  **Staff after commencing MAE x 24** |
|  | **Agitated** | 272 | Not agitated x 1  **Staff member** **x 2**  **Staff after commencing MAE x 2** |
|  | Shock (noun)  Shock (verb) | 32  18 | Physiological / anaphylactic / septic shock x 33 |
| **Sad** | Unhappy | 163 | **Staff member x 58**  **Staff after commencing MAE x 0** |
|  | **Sorry** | 34 | Unknown / used for describing the incident x 3  **Staff member x 24**  **Staff after commencing MAE x 13** |
| **Guilt** | **Fault** | 56 | **Staff member x 21**  **Staff after commencing MAE x 14** |
|  | Faulty | 55 | Device/ pump / insulin pen / cannula x 48 |
| **Depression** | Depression | 61 | **Staffs depressed after MAE x 0** |
|  | Frustrated | 26 | **Staff member x 3**  **Staff after commencing MAE x 0** |
| **Total** | 16 | **1861** | Staff members’ emotions **154**  **Staff members’ emotions after commencing MAE = 93** |

**Supplementary file_2.** Number of negative emotional expressions related specifically to medication administration incident reports (n=72,390)

| **Negative emotions** | **Synonyms of the terms** | **Number of expressions** | **Negative emotions** | **Synonyms of the terms** | **Number of expressions** |
| --- | --- | --- | --- | --- | --- |
| Fear / Anxiety | scare  fear  frighten/ -ed  frightful  fright  **worry**  **worried**  worry (noun)  terror  **anxiety**  **anxious**  nervous  doubt  mistrust  panic  nervousness  afraid  flashback  **stress / -ed (verb)**  **stress (noun)**  terrified  petrified  unsettled  on edge  **distressed**  jittery  fidgety  restless  uneasy  dread  apprehension  trepidation  **concerned**  uneasiness | 9  13  11  0  1  33  99  14  0  59  124  8  16  0  29  0  5  33  47  30  0  0  24  0  104  1  0  49  4  0  1  0  209  0 | Anger | **angry**  anger  furious  livid  pissed off  **annoy / annoyed**  miffed  bitter  enraged  exasperated / -ion  fuming  irate  incensed  antagonize  displeasure  aggravate  aggravation  huff  crossness  bile  spleen  indignation  displeasure  dudgeon  sullen  sulky | 91  3  2  0  0  23  0  0  0  0  0  4  0  0  2  2  0  0  0  12  2  0  2  0  0  0 |
| Upset | **upset**  disturb  **shock (noun)**  **shock (verb)**  shake  discompose  unhinge  stunned  unbalance  uneasy  solicitous  disappoint  **agitated** | 353  33  32  18  43  0  0  0  1  4  0  5  272 | Sad | sad  sorrowful  **unhappy**  **sorry**  mournful  rueful  distressing  lugubrious  woefully  afflicted  woe some  wretched  miserable  nasty  lousy  crappy | 1  0  163  34  0  0  0  0  1  0  0  0  1  2  0  0 |
| Shame | shame  ashamed  disgrace  ignominy  stigma  mortification  mortified  reproach  dishonor / dishonour  indignity  discredit  obloquy  embarrassed  embarrassing  shamefaced  hangdog  self-conscious | 0  0  0  0  0  0  0  0  0  0  0  0  0  3  0  0  0 | Guilt | guilt  guiltiness  self-blame  regret  regretful  offensive  **fault**  **faulty**  failing  culpability  self-reproach  blameworthiness  blame  wrongdoing  misconduct  self-reproach  self-condemnation  remorse  remorsefulness  remorseful  contrition  contriteness  compunction | 0  0  0  1  2  8  56  55  11  0  0  0  9  1  1  0  0  1  0  0  0  0  0 |
| Self-esteem | self-worth  self-regard  self-respect  self-integrity  self-confidence  self-disappointment  incompetent | 0  0  0  0  0  0  3 | Depression | **depression**  depress / -ed  **frustrate / -ed**  overwhelmed  devastate/ -ed  hopelessness  sleepiness  crestfallen  hump | 61  10  26  3  3  1  1  0  0 |

**Supplementary file_3.** SRQR checklist for reporting qualitative studies

| **No.** | **Standards for Reporting Qualitative Research (SRQR)*** | **Yes/No/Not applicable**  **Page no** |
| --- | --- | --- |
| **Title and abstract** | |  |
| **S1** | **Title** - Concise description of the nature and topic of the study Identifying the study as qualitative or indicating the approach (e.g., ethnography, grounded theory) or data collection methods (e.g., interview, focus group) is recommended | No  1 |
| **S2** | **Abstract** - Summary of key elements of the study using the abstract format of the intended publication; typically includes background, purpose, methods, results, and conclusions | Yes  1-2 |
|  |  |  |
| **Introduction** | |  |
| **S3** | **Problem formulation** - Description and significance of the problem/phenomenon studied; review of relevant theory and empirical work; problem statement | Yes  3-5 |
| **S4** | **Purpose or research questio**n - Purpose of the study and specific objectives or questions | Yes  5 |
|  |  |  |
| **Methods** | |  |
| **S5** | **Qualitative approach and research paradigm** - Qualitative approach (e.g., ethnography, grounded theory, case study, phenomenology, narrative research) and guiding theory if appropriate; identifying the research paradigm (e.g., postpositivist, constructivist/ interpretivist) is also recommended; rationale** | Not applicable |
| **S6** | **Researcher characteristics and reflexivity** - Researchers’ characteristics that may influence the research, including personal attributes, qualifications/experience, relationship with participants, assumptions, and/or presuppositions; potential or actual interaction between researchers’ characteristics and the research questions, approach, methods, results, and/or transferability | Not applicable |
| **S7** | **Context** - Setting/site and salient contextual factors; rationale** | Yes  6 |
| **S8** | **Sampling strategy** - How and why research participants, documents, or events were selected; criteria for deciding when no further sampling was necessary (e.g., sampling saturation); rationale** | Not applicable |
| **S9** | **Ethical issues pertaining to human subjects** - Documentation of approval by an appropriate ethics review board and participant consent, or explanation for lack thereof; other confidentiality and data security issues | Yes  8 |
| **S10** | **Data collection methods** - Types of data collected; details of data collection procedures including (as appropriate) start and stop dates of data collection and analysis, iterative process, triangulation of sources/methods, and modification of procedures in response to evolving study findings; rationale** | Yes  6 |
| **S11** | **Data collection instruments and technologies** - Description of instruments (e.g., interview guides, questionnaires) and devices (e.g., audio recorders) used for data collection, if/how the instrument(s) changed over the course of the study | Not applicable |
| **S12** | **Units of study** - Number and relevant characteristics of participants, documents, or events included in the study; level of participation (could be reported in results) | Not applicable |
| **S13** | **Data processing** - Methods for processing data prior to and during analysis, including transcription, data entry, data management and security, verification of data integrity, data coding, and anonymization/de-identification of excerpts | Yes  6-8 |
| **S14** | **Data analysis** - Process by which inferences, themes, etc., were identified and developed, including the researchers involved in data analysis; usually references a specific paradigm or approach; rationale** | Yes  6-8 |
| **S15** | **Techniques to enhance trustworthiness** - Techniques to enhance trustworthiness and credibility of data analysis (e.g., member checking, audit trail, triangulation); rationale** | Yes  8 |
|  |  |  |
| **Results/findings** | |  |
| **S16** | **Synthesis and interpretation** - Main findings (e.g., interpretations, inferences, and themes); might include development of a theory or model, or integration with prior research or theory | Yes  9-17 |
| **S17** | **Links to empirical data** - Evidence (e.g., quotes, field notes, text excerpts, photographs) to substantiate analytic findings | Yes  8-12 |
|  |  |  |
| **Discussion** | |  |
| **S18** | **Integration with prior work, implications, transferability, and contribution(s) to the field -** Short summary of main findings; explanation of how findings and conclusions connect to, support, elaborate on, or challenge conclusions of earlier scholarship; discussion of scope of application/generalizability; identification of unique contribution(s) to scholarship in a discipline or field | Yes  12-14 |
| **S19** | **Limitations** - Trustworthiness and limitations of findings | Yes  14-15 |
|  |  |  |
| **Other** | |  |
| **S20** | **Conflicts of interest** - Potential sources of influence or perceived influence on study conduct and conclusions; how these were managed | Yes  Title page |
| **S21** | **Funding** - Sources of funding and other support; role of funders in data collection, interpretation, and reporting | Yes  Title page |
|  |  |  |
| *The authors created the SRQR by searching the literature to identify guidelines, reporting standards, and critical appraisal criteria for qualitative research; reviewing the reference lists of retrieved sources; and contacting experts to gain feedback. The SRQR aims to improve the transparency of all aspects of qualitative research by providing clear standards for reporting qualitative research. | | |
| **The rationale should briefly discuss the justification for choosing that theory, approach, method, or technique rather than other options available, the assumptions and limitations implicit in those choices, and how those choices influence study conclusions and transferability. As appropriate, the rationale for several items might be discussed together. | | |
| **Reference:** | | |
| O'Brien BC, Harris IB, Beckman TJ, Reed DA, Cook DA. (2014). Standards for reporting qualitative research: a synthesis of recommendations. *Academic Medicine*, 89 (9),1245-1251.  DOI: 10.1097/ACM.0000000000000388 | | |
